# The hydrodynamic performance of duck feet for submerged swimming resembles oars rather than delta-wings

**DOI:** 10.1038/s41598-023-42784-w

**Published:** 2023-09-27

**Authors:** Gal Ribak, Roi Gurka

**Affiliations:** 1 School of Zoology, Faculty of Life Sciences, Tel Aviv University, 6997801 Tel Aviv, Israel; 2 The Steinhardt Museum of Natural History, Tel Aviv, Israel; 3 Physics and Engineering Science, Coastal Carolina University, Conway, SC USA

**Keywords:** Biomechanics, Aerospace engineering

## Abstract

Waterfowl use webbed feet to swim underwater. It has been suggested that the triangular shape of the webbed foot functions as a lift-generating delta wing rather than a drag-generating oar. To test this idea, we studied the hydrodynamic characteristics of a diving duck’s (*Aythya nyroca*) foot. The foot’s time varying angles-of-attack (*AoA*s) during paddling were extracted from movies of ducks diving vertically in a water tank. Lift and drag coefficients of 3D-printed duck-foot models were measured as a function of *AoA* in a wind-tunnel; and the near-wake flow dynamics behind the foot model was characterized using particle image velocimetry (PIV) in a flume. Drag provided forward thrust during the first 80% of the power phase, whereas lift dominated thrust production at the end of the power stroke. In steady flow, the transfer of momentum from foot to water peaked at 45° < *AoA* < 60°, due to an organized wake flow pattern (vortex street), whereas at *AoAs* > 60° the flow behind the foot was fully separated, generating high drag levels. The flow characteristics do not constitute the vortex lift typical of delta wings. Rather, duck feet seem to be an adaptation for propulsion at a wide range of *AoAs*, on and below the water surface.

## Introduction

Avian divers needed to solve the biomechanical conflict between their primary adaptation, aimed towards flight, and their secondary adaptation, for swimming underwater. The first conflict ensues from the low density of the avian body (due to the hollow bones, large air sacs, and layer of air trapped under the waterproof plumage), resulting in a high buoyancy that resists diving by pushing the diver upward towards the surface^1–3^. Consequently, many waterfowl swim on the water surface, but only a few are also adapted to swimming underwater. The second conflict involves the use of wings for propulsion in both air and water, which differ ~ 850-fold and ~ 55-fold in their density and viscosity, respectively, making large wings inefficient underwater and small wings inefficient for flight. The compromise of using the same wing for flying and swimming entails a toll on the efficiency of at least one of these forms of locomotion^4,5^. Most waterfowl avoid the second conflict by keeping their wings adapted for flight and swim exclusively using their webbed feet while the wings remain folded close to the body (but see Richman and Lovvorn^6^, for combined wing and foot propulsion underwater).

Because the paddling feet move backwards relative to the body during the power phase, foot propulsion has been considered to be a form of drag-based propulsion^7^. ‘Drag-based’ propulsion implies that the forward thrust propelling the animal forward is dominated by the drag of the swimming appendages (fins, feet, etc.) whereas in ‘lift-based’ propulsion the lift forces generated by the swimming appendages dominate forward thrust production. The moving appendage can generate both lift and drag but these forces are perpendicular to each other; therefore, their contribution to forward thrust depends on the kinematics of the appendage and its shape^8^. In some cases both lift and drag can contribute to propulsion either together or at different parts of the power phase^9^.

Drag-based propulsion is less efficient when the speed of swimming increases^10^, favoring a transition in semi-aquatic animals from drag-dominated to lift-dominated propulsion according to their level of adaptation to an aquatic lifestyle ^11,12^. Studies on the foot motions of fast- underwater avian swimmers such as cormorants and grebes have shown that the feet move relative to the water in a trajectory that mostly generates hydrodynamic-lift force for forward thrust^13–15^. The triangular webbed foot has a low aspect-ratio that does not seem ideal for lift production. However, it was suggested that a triangular planform can function similarly to a delta wing^14^. Delta wings are triangular, low-aspect ratio wings that generate substantial lift due to a vortex generated on the swept, sharp, leading-edge^16^. Delta wings are less prone to stall at a large angle-of-attack (*AoA*) ^17^—a property that could benefit swimming animals that oscillate their feet or fins at large amplitudes during swimming. Alternatively, a triangular appendage oscillated by its apex is an efficient drag-producing appendage^18^ because both the tangential speed and area of the swinging appendage increase with distance from the apex. Consequently, the faster moving and broader base of the trailing edge affects a greater mass of water thus generating most of the drag used as propulsive force^18,19^. The hydrodynamics of the avian webbed foot has been previously studied with respect to drag-based vortex formation on rigid plates^20^ or as a flexible flipper^21,22^ for biomimetic surface swimming, hydroplaning and takeoff from water^23–26^. However, the hydrodynamic functioning of the foot while swimming underwater should differ from these conditions at the water surface due to differences in paddling kinematics, body orientation and the need to resist buoyancy when the body is entirely submerged.

The magnitude of the lift generated by the motion of a foot depends on the foot’s velocity, area, and *AoA*. The direction of this lift force is perpendicular to the velocity of the foot relative to water, which in turn is determined by the combination of the backwards motion of the foot relative to the body (paddling) and the forward velocity of the body (swimming speed). Swimming at high velocity leads to the small *AoA*s that are adequate to generate lift. This lift is oriented with a large horizontal component, delivering thrust to propel the body forward during horizontal swimming^14,15^. However, it is unknown to what extent slow-swimming divers (such as ducks) are capable of generating similar lift forces with their webbed feet during swimming.

Diving ducks mainly perform vertical foraging dives from the water surface to consume food located in the benthos, and then return to the surface. While holding their position to feed next to the bottom (zero mean swimming speed), their foot propulsion is entirely drag-based^27^. The ascent from the bottom back to the surface is entirely passive, exploiting the ducks’ buoyancy. In contrast, the descent from the surface to the bottom requires that the ducks swim against buoyancy, slowing down their descent.

We examined how the duck’s foot motion interacts with the low swimming speeds during such descent to change the foot’s *AoA*, and tested the hydrodynamic properties of the webbed foot over a range of *AoA*s in the contexts of the steady lift and drag it generates and the flow generated in its near wake. The study incorporated filming the paddling motions of captive diving ducks as they descended towards the bottom in vertical dives. From the movies we extracted the foot kinematics during the power phase, focusing on the instantaneous changes of the *AoA*. Next, we printed 3D models of the webbed foot in order to study its hydrodynamic characteristics. We measured the lift and drag coefficients as a function of the foot’s *AoA* in a wind tunnel, and analyzed the interaction of the foot with the surrounding fluid in a water flume using particle image velocimetry (PIV).

## Results

### Paddling kinematics

**Figure 1:**
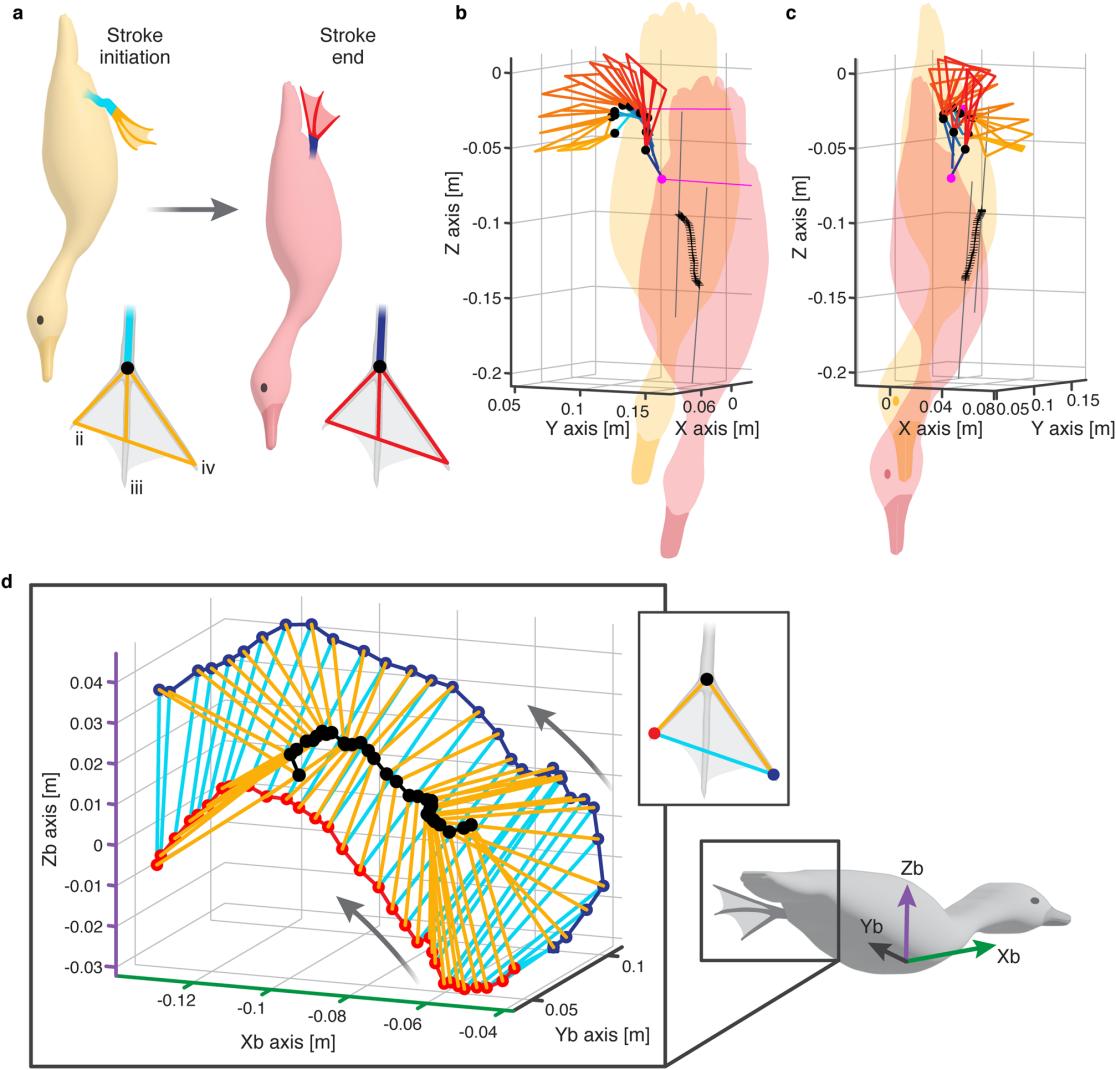
Paddling kinematics at 1.5 m depth during the descent of a diving duck (*A. nyroca*) to the bottom. (**a**) The orientation of the body and the position of the left foot at the power phase initiation and termination, as well as a definition of the planform of the foot as measured from points in the movies (see Fig. 10 in the methods). Roman numerals denote the anatomical digit (toe) number for avian feet. The kinematics of the paddling feet is shown in ventral (**b**) and side (**c**) view, with the color gradient from orange to red corresponding to the advancement in time between initiation and termination of the power phase as depicted in (**a**). For improved clarity, the foot kinematics data in (**b**) and (**c**) are depicted every fourth video frame (i.e., interspaced by 16 ms). Z is the vertical axis of the diving tank with the positive end pointing up. Black crosses denote the instantaneous positions of the body (halfway between the neck and tail) of the downwards-swimming duck. Magenta lines connect the Tarsus-tibia joints of the left and right legs at the power stroke initiation and termination. (**d**) The position and orientation of the left foot in the body frame of reference during the power phase of one paddling cycle. Xb is the longitudinal body axis (positive towards the head), Yb is the lateral body axis (positive towards the left side), and Zb is the dorsoventral axis (positive towards the dorsal side) as illustrated on the duck image on the right. Red and blue circles denote the position of the tip of the interior (ii) and exterior (iv) digits (F_2_ and F_3_), respectively. Black circles represent the inter-digit joint (F_1_). Orange and cyan lines denote the digits and foot span, respectively. The data are shown for each movie frame (interspaced by 4 ms). Arrows show the direction of motion from the start of the power phase. Note that the foot is moving mostly medially during the last 20% of the power phase duration.

We collected data from a total of 22 paddling cycles in 11 movies showing ducks diving vertically to the bottom of a 2 m deep tank. Each movie showed at least one foot during the power phase of the paddling cycle as the ducks approached 1.5 m depth. At that depth, the ducks were swimming towards the bottom at a mean (± SD) speed of 0.32 ± 0.137 m s^−1^ and paddling their feet at 4.3 ± 0.43 Hz. Figure 1a–c presents an example of the 3D paddling kinematics in the cameras’ frame of reference. The ducks descended the water column head first, sweeping their feet in a curvilinear trajectory with a large vertical (Z axis) component. The mean excursion of the foot perpendicular to the swimming direction (in the XY plane) during the power stroke was 0.035 ± 0.012 m yielding Strouhal number (paddling frequency $\times$ amplitude/swimming speed) of St = 0.47.

**Figure 2:**
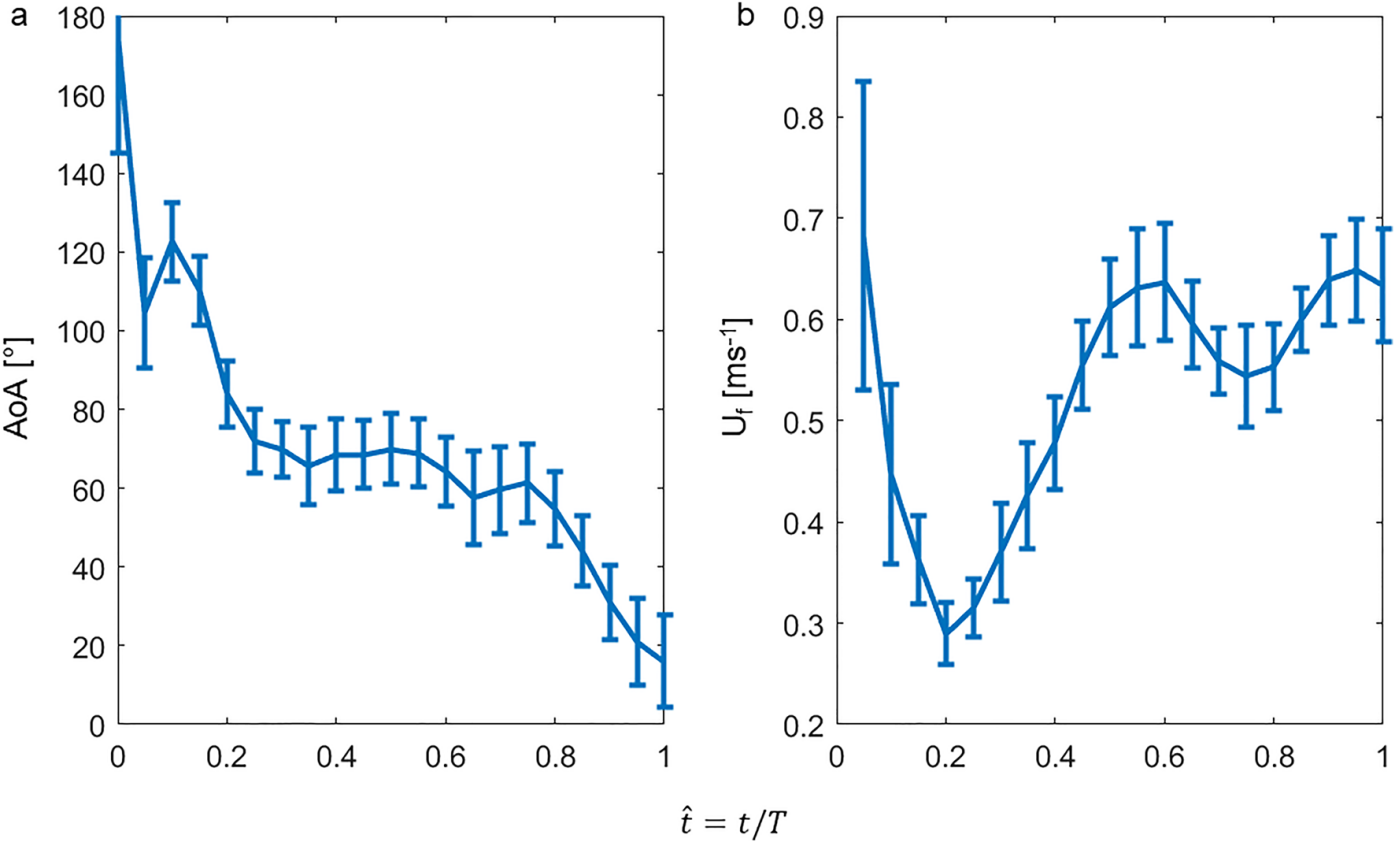
(**a**) Change in *AoA* during the power phase of a paddling cycle. The data are averaged over 22 paddling cycles. Error bars denote SE. b) The speed of the foot relative to the water measured at point F_5_ (2/3 along the central chord, see Fig. 10). The horizontal axis in both (**a**) and (**b**), is the normalized time within the power phase, i.e., *t/T* where *t* is time and *T* the power phase duration.

**Figure 3:**
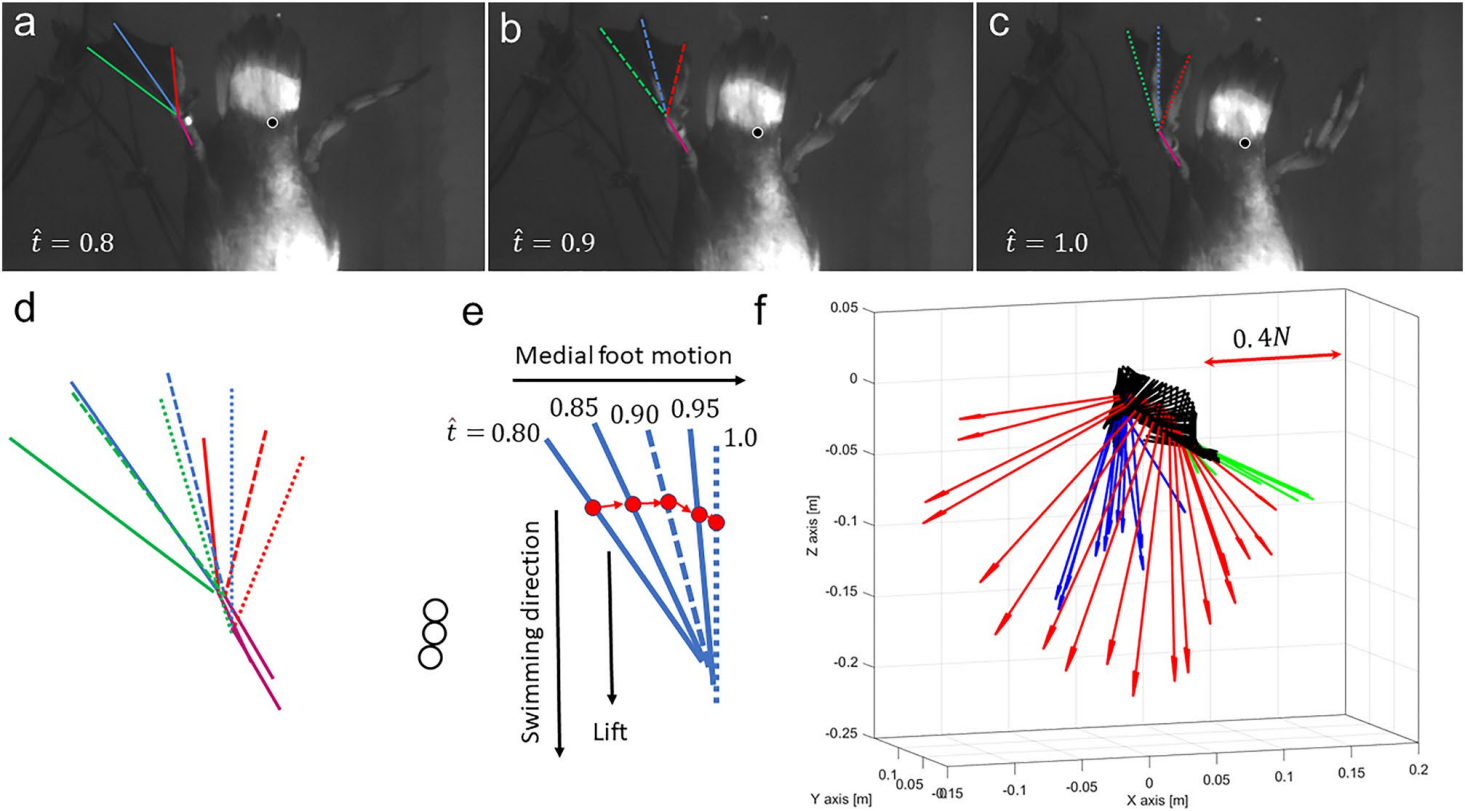
(**a**–**c**) The orientation and motion of the left foot (relative to the water) during the last 20% of the power phase (0.8 < $\widehat{t}$<1.0). Digits ii, iii, and iv are marked in red, blue, and green lines, respectively. Magenta color denotes the metatarsus. The circles in (**a**–**c**) track the vertical motion (descent) of the duck’s body. d) The same lines and circles in (**a**–**c**) are overlaid atop one another to emphasize the motion of the body and the flexion of the digits relative to the metatarsus at $\widehat{t}$ =0.8 (solid lines), 0.9 (dashed lines), and 1.0 (dotted lines). (**e**) Digit iii, showing the medial trajectory of point F_5_ at two thirds the chord of the foot (red circles) resulting in an oblique AoA. (**f**) An estimate of the quasi-steady hydrodynamic force generated by the foot during the power phase. The position and orientation of the webbed area of the left foot relative to the water is shown at each 4 ms of the power phase duration as black triangles. Arrows are the corresponding estimates of the resultant force (lift + drag) based on the foot’s *AoA*, area, and velocity, derived from the high-speed movie and the force coefficients found in the wind-tunnel. Green, red, and blue arrows denote the resultant force vectors at the first 20%, the next 60%, and the last 20% of the power phase, respectively. The forces are calculated for the left foot, then multiplied by two to account for both feet paddling simultaneously. Arrows lengths denote the estimated force magnitude in N, according to the scale bar shown in the upper right corner.

Figure 1d presents the foot’s trajectory and orientation in the duck’s frame of reference. The foot moved both backwards, medially, and dorsoventrally relative to the body, but in the final 20% of the power phase the motion was only medial. The interaction of this curvilinear trajectory with the descending speed of the body resulted in an instantaneous foot *AoA* and velocity that varied over the power phase duration (Fig. 2). At the power phase initiation (0 < $\widehat{t}$<0.2 in Fig. 2), the forward-oriented digits moved relative to the water at an obtuse *AoA* with the inter-digit apex of the triangular foot (point F_1_) trailing. The foot moved at low speed because as it started to move backwards relative to the body, the body moved forward (descending in the water column, Fig. 1) such that the two opposing velocity vectors resulted in a low-speed relative to the stagnant water. This paddling initiation phase is thus not expected to result in substantial lift and drag. Then, as the foot swept mostly backwards (0.2 < $\widehat{t}$<0.8) with the apex of the foot leading, it reached a steady *AoA* =  ~ 70°, while the foot velocity increased relative to the water. At $\widehat{t}$=0.8 the foot transitioned from moving backwards, laterally and towards the dorsal side relative to the body, to its medial motion at an oblique *AoA,* with some downwards movement relative to the water (Fig. 3). At this stage the foot speed remained at its top speed relative to the water (Fig. 2b) and the low *AoA* can be used to generate lift. The digits-tarsus joint starts to flex but the webbed area remains large (Fig. 3a–d). The lift generated by the low *AoA* and the lateral movement will be directed downwards (i.e., forward along the body’s longitudinal axis, Fig. 3e,f) helping to propel the body forward against drag and buoyancy.

### Measurements of hydrodynamic forces

**Figure 4:**
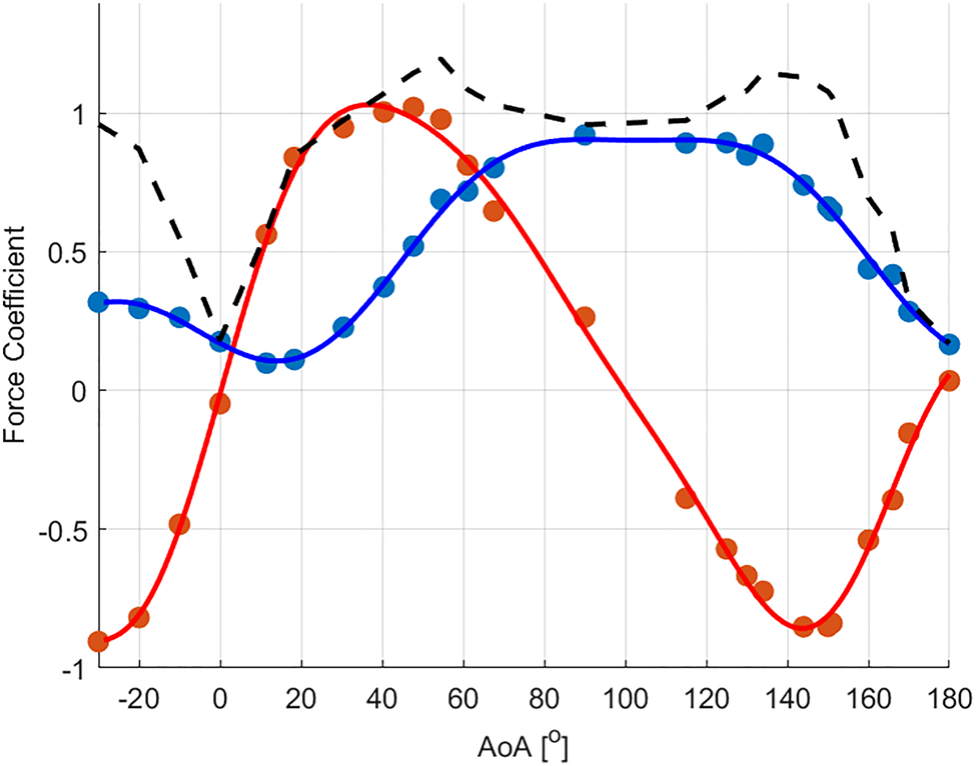
Hydrodynamic performance of the duck-foot model. Wind tunnel results showing the lift (red circles) and drag (blue circles) coefficients as a function of *AoA* at Reynolds number Re = 31,000. Dashed black line denotes the resultant force coefficient (${\mathrm{C}}_{\mathrm{F}}=\sqrt{{\mathrm{C}}_{\mathrm{D}}^{2}+{\mathrm{C}}_{\mathrm{L}}^{2}}$). The relationships between the lift and drag coefficients and the *AoA* can be approximated (solid lines) by the sum of sine functions (${\sum }_{i=1}^{n}{A}_{i}\mathrm{sin}\left({b}_{i}\alpha +{\varphi }_{i}\right)$: ${C}_{L(\alpha )}=0.72\mathrm{sin}\left(2\alpha +0.35\right)+14.8\mathrm{sin}\left(5.47\alpha -1.6\right)+14.8\mathrm{sin}\left(5.5\alpha +1.5\right)+0.49\mathrm{sin}\left(1.5\alpha -0.4\right)$. ${C}_{D(\alpha )}=0.73\mathrm{sin}\left(0.6\alpha +0.48\right)+0.25\mathrm{sin}\left(2.23\alpha +3.86\right)+0.09\mathrm{sin}\left(4.35\alpha +3.19\right)$ where $\alpha$ is the *AoA* in radians.

We mounted a 3D printed duck-foot model in a wind tunnel and measured its lift and drag coefficients as a function of *AoA* with force transducers (Fig. 4). The drag exhibits a sigmoidal increase between 10° and 90°. At obtuse *AoA*s, the drag does not change up to 140°, followed by a monotonic decrease to 180°. The lift coefficient increases linearly between 0° and 20°, (d*C*_*L*_/d*AoA* = 0.042 1/°), and then increases non-linearly to *C*_Lmax_ = 1.02 at 45°. The lift values are the highest between 30° and 50° and then decline up to 140°. At *AoA* > 90° the apex of the triangular foot is facing downstream and the lift becomes negative, as expected for these *AoA*s. The lift trend at obtuse *AoA*s differs from that at *AoA* < 90°, demonstrating an asymmetric hydrodynamic load effect between apex-first or base-first motion of the foot. The lift-to-drag ratio reaches a maximum at *AoA* =  ~ 25° and is greater than 1 at 10° < *AoA* < 60° and smaller than 1 between 60° < *AoA* < 130°, as expected for pre-stall and fully separated flow, respectively. As a result, the magnitude of the resultant hydrodynamic force generated by the foot (${\mathrm{C}}_{\mathrm{F}}=\sqrt{{\mathrm{C}}_{\mathrm{D}}^{2}+{\mathrm{C}}_{\mathrm{L}}^{2}}$) reached a maximum at *AoA* = 55° and remained almost unchanged in magnitude over a wide range of *AoA*s (40°–150^o^).

### Mean wake flow characteristics

The wind tunnel experiments provided an integral overview of the forces exerted by the duck foot. To complement the study with a differential overview of the wake we placed a 3D printed duck-foot model in a water flume at different *AoA*s and measured the flow fields in the foot’s wake. 

#### Near-wake flow dynamics

**Figure 5:**
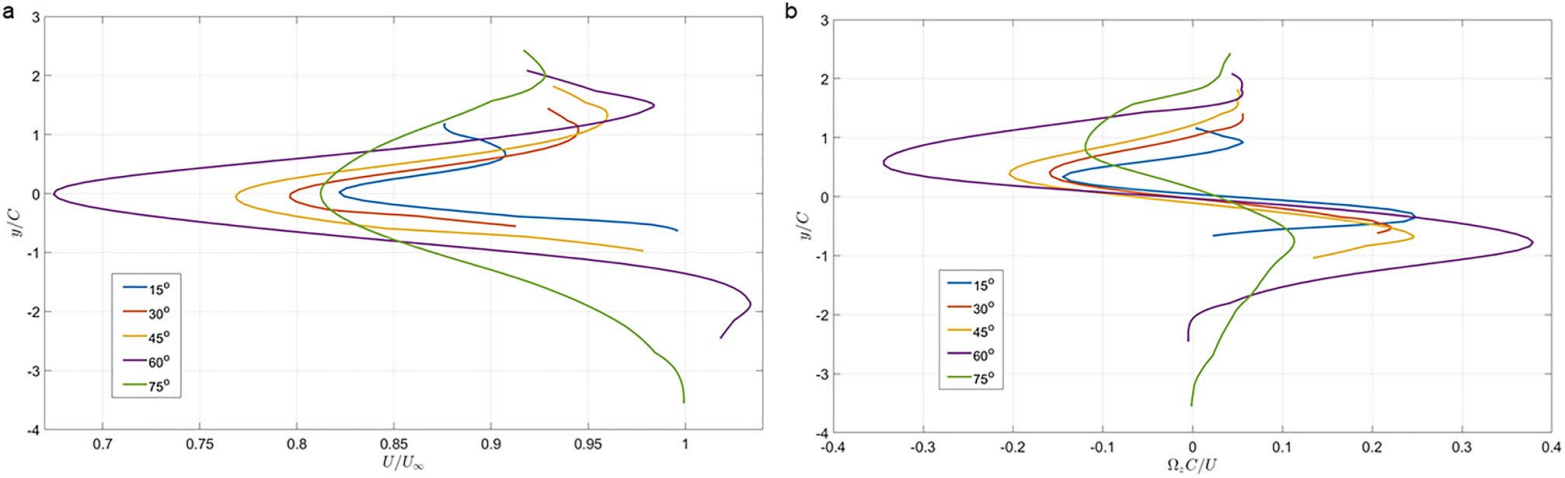
Near-wake flow characteristics: (**a**) mean streamwise velocity profiles; (**b**) mean spanwise vorticity profiles. Profiles were averaged over 500 vector maps and two chord lengths (2*C*) in the streamwise direction. ${U}_{\infty }$ is the free stream velocity and ${\Omega }_{2}$ is the mean spanwise vorticity.

Hydrofoil wake is commonly associated with the decrease in flow speed (‘deficit’) which is proportional to the drag it generates^28^. The mean streamwise velocity profile of the near-wake region of the duck foot (Fig. 5a) exhibited an increased deficit as a function of the *AoA*. The largest deficit was observed at *AoA* = 60°. At *AoA* = 75°, the flow was presumably fully separated, causing the wake to expand and capture a wider region within the flow, as indicated by the expansion of the green curve towards $y/C=$ 4 in Fig. 5a. Consequently, integration of the area under the curve will yield a larger deficit (higher drag) compared to cases in which *AoA* < 60°. The velocity profile formed behind the foot is proportional to the boundary layer formed over the foot at a specific spanwise location; thus, the thickness of the foot’s boundary layer increases up to 60°, causing changes in the wake velocity profile and its gradients. The mean spanwise vorticity (Fig. 5b) results from the incoming shear layers associated with the fluid-foot interaction, and changes with the *AoA* in conjunction with the differences observed in the streamwise velocity profiles. The spanwise vorticity thus depends strongly on the change in the streamwise velocity in the normal direction. The trend observed is thus a derivative of Fig. 5a, i.e.: a positive and a negative peak marking the entrainment region between the wake and the free stream region. The increasing peak values up to 60° represent the strengthening of the entrainment regions within the wake region. At 75°, where the flow is fully separated over the foot, the region behind the foot is not dominated by wake characteristics.

**Figure 6:**
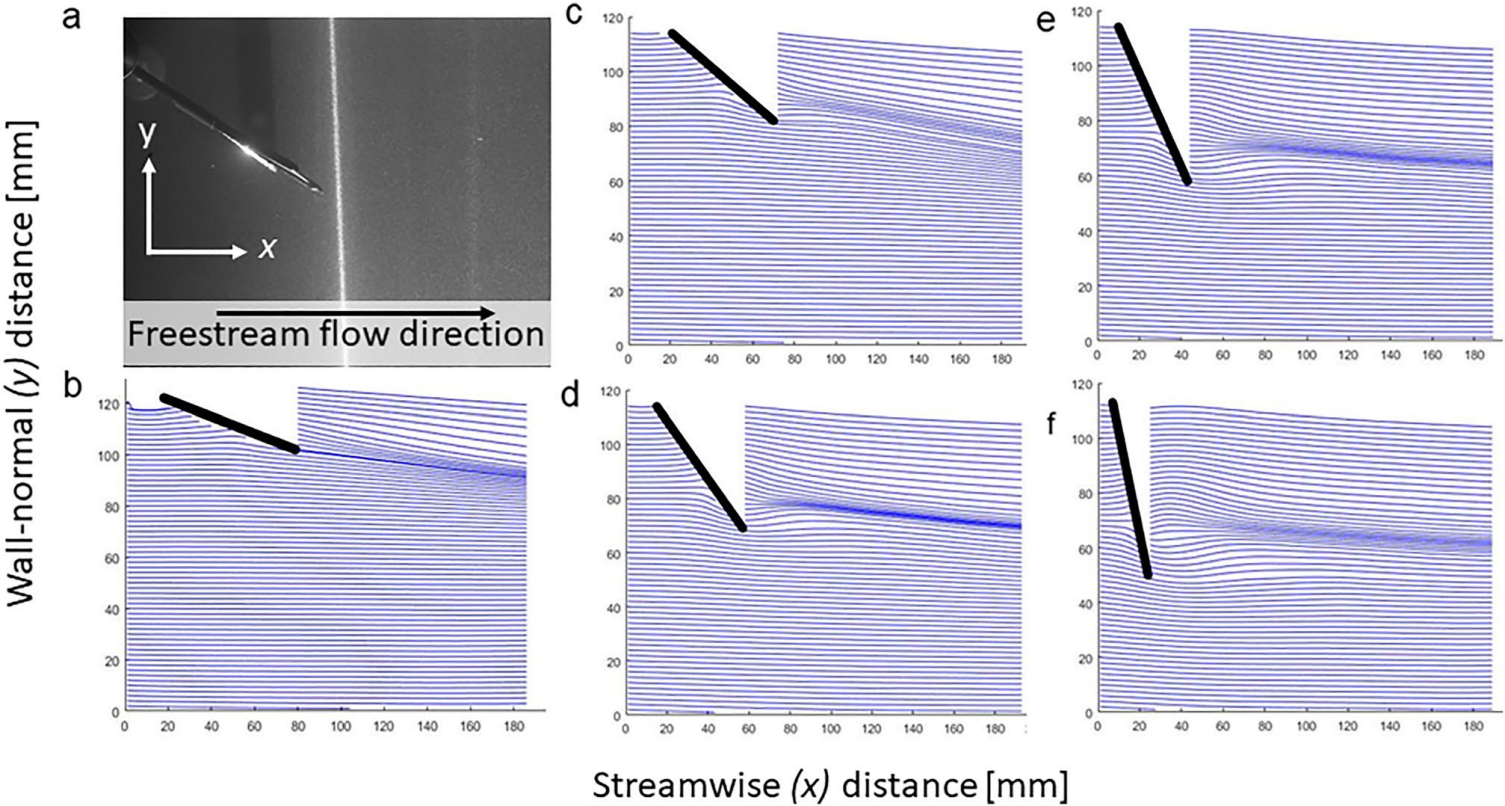
Streamlines of the mean velocity field in the near-wake region of the duck foot. (**a**) the foot model illuminated by the laser sheet in the flume and the definitions of the streamwise and wall-normal directions, (**b**) *AoA* = 15°, (**c**) *AoA* = 30°, (**d**) *AoA* = 45°, (**e**) *AoA* = 60°, and (**f**) *AoA* = 75°. Note that these streamlines are calculated based on the ensemble mean velocity field and therefore depict the flow deflection due to the foot but conceal dynamic phenomena such as vortex shedding or separation.

To acquire a qualitative and more spatially-explicit insight into the wake flow patterns, we first calculated the streamlines (Fig. 6) in the two-dimensional (vertical) plane as $dy/dx=v/u$. The streamlines of the flow were computed for the mean velocity maps of each *AoA*. As such, they do not describe an instantaneous description (i.e.: flow separation) or visualize any shedding but rather the incident (downwards momentum) of the mean flow at the trailing edge of the foot and over its suction surface. At *AoA* < 45°, the streamlines follow the change in flow in respect to the *AoA*. Above 45°, the streamlines exhibit some sort of deflection resulting from the blockage effect formed by the foot at high *AoA*s (Fig. 6d–f).

#### Wake turbulence characteristics

**Figure 7:**
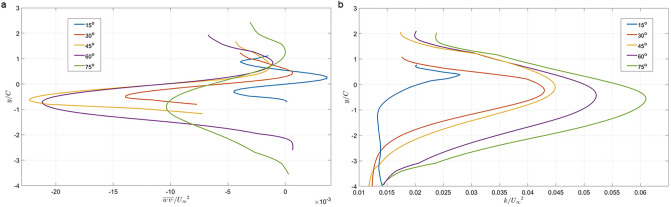
(**a**) Reynolds stress; and (**b**) turbulent kinetic energy in the near-wake of the duck-foot model at different *AoA*s (line colors). Turbulence properties were averaged over 500 vector maps and two chord lengths (2*C*) in the streamwise direction. ${U}_{\infty }$ is the free stream velocity.

Hydrodynamic forces at low-intermediate Reynolds (Re) numbers are determined by the wake dynamics, which is dependent on the turbulence characteristics. The change in turbulence in the wake as a function of *AoA* was evaluated by calculating the vertical profiles of Reynolds stress ($\overline{{u }{\prime}{v}{\prime}}$) and of the 2D turbulent kinetic energy (TKE = $0.5\left(\overline{{u }^{{\mathrm{^{\prime}}}^{2}}}+\overline{{v }^{{\mathrm{^{\prime}}}^{2}}}\right))$ in the near wake for each *AoA*. Both were averaged over two chord lengths in the streamwise direction and normalized by the mean streamwise velocity. The trends for both turbulence quantities (Fig. 7) are similar over the range of *AoA*s. Reynolds stress changes across the wake in a non-symmetrical fashion, affected by the *AoA*. Similar trends can be observed in cases where the Reynolds stress value peaks in the wake region and decreases with distance from the wake. The high values obtained for 60° and 75° are more than double those of lower angle cases. The Reynolds stress presents a similar trend to that of the spanwise vorticity^29^. TKE mirrors the dependency of Reynold stresses on *AoA*, since both increase as a function of *AoA*, suggesting higher turbulence activity at high *AoA*s. However, unlike Reynolds stress, here the increase is gradual. The dramatic change in Reynolds stress between 30° and 45° is presumably associated with a change in the wake flow structure that is aligned with the increase in drag and maximal lift of the hydrofoil (Fig. 4).

#### Near-wake flow patterns

Finally, the turbulence in the wake can be visualized using proper orthogonal decomposition (POD)^30,31^ applied to the PIV velocity data^32^. POD transformation enables an orthonormal projection of the flow quantities onto a minimal number of uncorrelated modes^31^. It aims to describe coherent patterns in a given flow field, if such exist. The presence of such patterns indicates a constructive momentum transfer (thrust) in between the foot and water. The description is commonly arrived at through morphological characteristics. Conceptually, applying POD to the velocity field is akin to decomposing the field into energetic (kinetic) modes, where the energy distribution is ranked from low to high. We reconstructed the data for all cases based on the first 100 modes, which account for about 92% of the total energy. The total energy contained by the modes is the highest at 15° and then decreases up to 90° where the trends flip when the angles become greater than 90° (Supplementary Figure 1). This observation is in line with that of the drag formed over the feet increasing with the *AoA*, resulting in maximal energy loss due to blockage from the feet at 90°.

**Figure 8:**
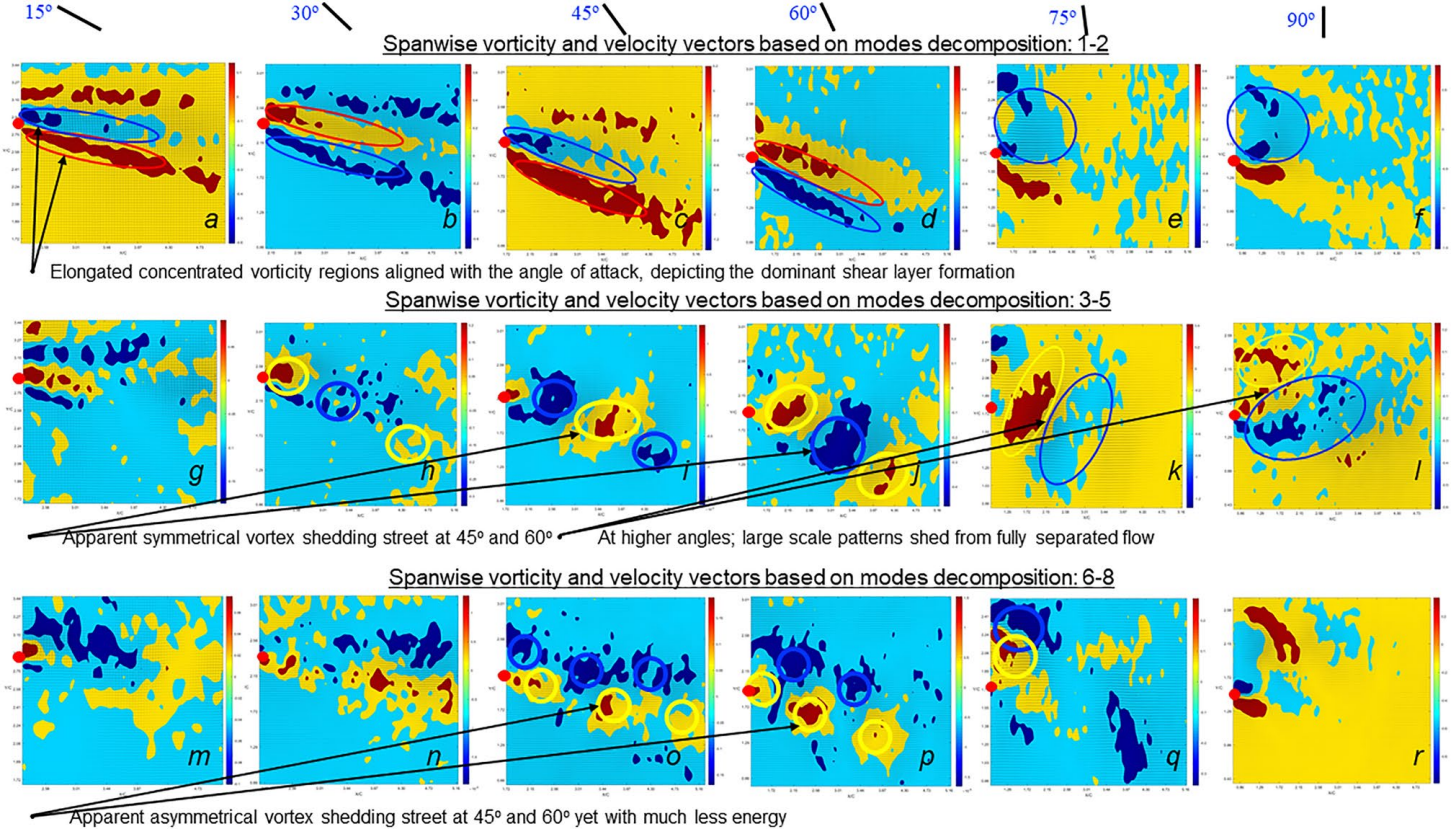
Spanwise vorticity and velocity vectors based on the decomposed velocity field using linear combination of the modes: Top modes 1 + 2, Middle modes 3 + 4 + 5, and Bottom modes 6 + 7 + 8, in the near-wake region of the duck feet. (**a**, **g**, **m**) *AoA* = 15°, (**b**, **h**, **n**) *AoA* = 30°, (**c**, **i**, **o**) *AoA* = 45°, (**d**, **j**, **p**) *AoA* = 60°, (**e**, **k**, **q**) *AoA* = 75°, and (**f**, **l**, **r**) *AoA* = 90°. Alternating positive and negative shed vortical structures are encircled by yellow and blue lines, respectively. The red dot marks the location of the foot trailing edge tip.

For all cases, the modes are presented by the velocity vectors (quiver plots) combined with the spanwise vorticity calculated for each mode. At higher modes, the numerical and experimental noise plays a significant role. Therefore, we focused only on the first eight modes in order to infer the fluid phenomena. Using linear combinations, we compacted these eight modes into three major groups: 1 + 2, 3 + 4 + 5, and 6 + 7 + 8, and plotted the three groups (image rows) as a function of *AoA* (columns) in Fig. 8. Modes 1 and 2 are associated with the mean flow characteristics. The intermediate modes (3:5) are presumably associated with the turbulence activity and thus bear the kinetic energy of the turbulent portion of the flow^33^. The higher modes (6:8) include mostly numerical and experimental error and are provided here merely for comparison with the lower modes. Because we focus on the geometrical variation of the flow across the mode groups, our main interest lies in the concentrated regions of normalized spanwise vorticity (positive and negative, normalized by the local mean vorticity). The first two modes show a strong shear pattern, resulting from a merging of the boundary layers shed at the suction and pressure sides of the foot, as expected in wake flows. These appear as a similar feature up to 60° for modes 1 + 2. This feature, marked as blue and red ellipsoids, is aligned with the *AoA* along the streamwise direction. Above this angle, the flow is presumably fully separated and, rather than organized wake, we can identify a sort of circular vorticity pattern that may be associated with a separation bubble.

The changes within the decomposed velocity field as a function of *AoA* at modes 3:5 depict a strong circular motion over the duck foot at the suction surface at *AoA* between 45° and 60°. At 15°, no shedding is observed. At 30°, circulatory motion is seen at the tip whilst at 75° and 90° the flow is presumably fully separated and a strong turbulence without any clear pattern can be identified. At 45° and 60° a symmetrical vortex street appears. At 30 ^o^ it also appears to form, but weaker. The presence of a vortex street indicates that at this *AoA* the flow constitutes an organized coherent structure, in which the lift will presumably increase^34^. This result explains Fig. 4, where the lift coefficient increases up to *AoA* = 45° and remains high at *AoA* = 60°*.* At higher angles (75° and 90°), a large circular motion can be identified, similar to modes 1 + 2, suggesting a fully separated flow without any lift production, but with high drag; which is in agreement with the maximum drag obtained at these angles (Fig. 4). At higher modes (6:8), the kinetic energy contribution is significantly smaller compared with the lower modes. However, at 45° and 60°, an asymmetric vortex street appears, which can be a consequence of the small-scale turbulence. For all other angles, we did not observe any significant geometrical pattern. This lack of geometrical pattern is common when decomposing a turbulent flow field at low to intermediate Re number, where there is almost no separation of scale, and energy transfer is limited.

## Discussion

The webbed feet of vertically diving ducks undergo large changes in their *AoA* during the power phase of paddling. During most of the power phase (at 0.2 < $\widehat{t}$<0.8) the feet move at a fairly constant *AoA* =  ~ 70°, which provides a similar (maximal) drag force to that of 90° but with a higher contribution of lift (Fig. 4). The term ‘lift force’ is used here in a broad sense to describe the hydrodynamic force component that is perpendicular to foot velocity and drag, rather than to indicate vortex flow circulation. During the two transient phases the foot is either at an obtuse *AoA* (power phase initiation) or at an oblique *AoA* (power phase termination). The foot’s triangular shape operates as a hydrofoil regardless of whether its apex is the leading or trailing edge. This phenomenon has been previously studied in delta wings. Although the ‘point-flying-last’ orientation has a large leading edge, its d*C*_*L*_/d* AoA* is typically smaller than the ‘point-flying-first’ orientation, indicating the importance of the trailing edge and wing sweep for vortex lift generation^17^. The foot model behaved similarly, with a shallower negative lift peak (*C*_*L* (*α* = 150_^o^_)_ = − 0.85) and smaller d*C*_*L*_/d* AoA* slope (0.024 *versus* 0.042).

**Figure 9:**
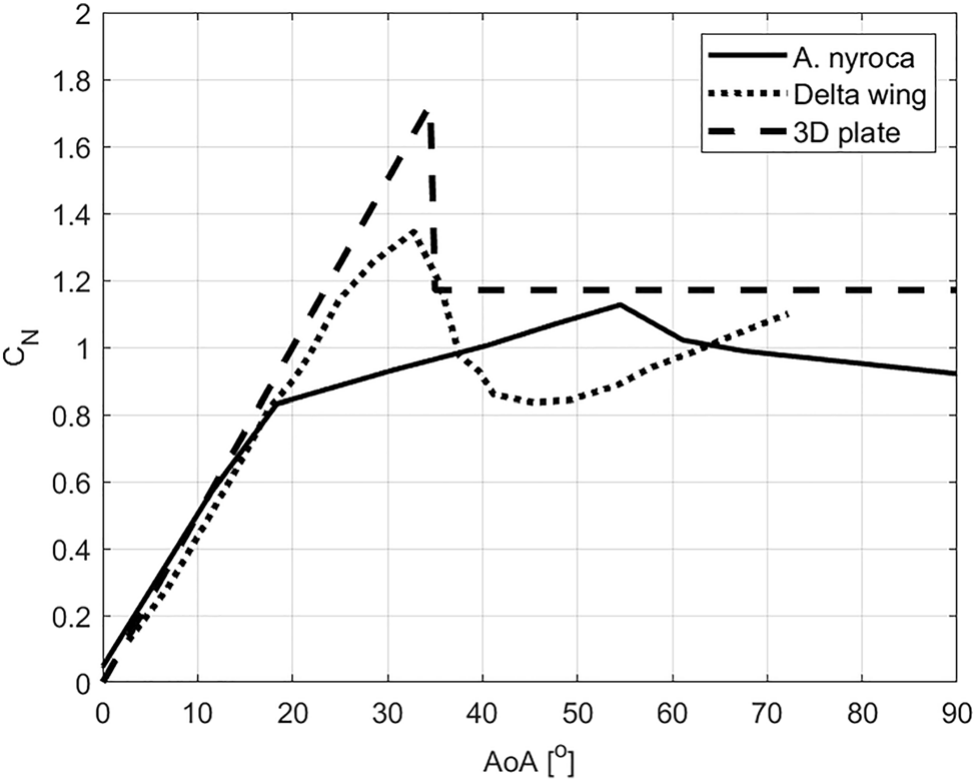
Normal force coefficients as a function of *AoA* for the duck-foot model and for published data on 60° delta wings^17^ and low aspect-ratio 3D plates^35^.

Hoerner^17^ noted that delta wings with an apex angle of 60° stall at ~ 33°, but continue to generate substantial lift up to ~ 45°, even after flow separation from the suction side. This is presumably enabled by residual low pressure remaining at the separated flow. While this vertical force can be considered a component of the pressure drag it is larger than expected for a flat plate, illustrating that some circulation remains at *AoA*, within a range of 30°–60°. In the foot of an *A. nyroca* duck the angle between digit ii and iv is 70°, and the shorter toe ii (compared to iv) contributes to narrowing of the foot planform (smaller chord) relative to the span. The effective *AR* is therefore higher than that of fully symmetric delta wings (isosceles triangle). We calculated the normal force coefficient (*C*_N_) for the duck-foot model from our wind tunnel data using the angle of the resultant force; ${\mathrm{tan}}^{-1}\left({C}_{L}/{C}_{D}\right)$ and the *AoA*, and compared it to Hoerner’s data compilation^17^ for delta wings with an apex angle of 60° (page 18–5, Fig. 7 in^17^), as well as to his data^35^ on 3D low-aspect ratio plates that generate vertical force from the cross-flow principle rather than from circulation (page 3–16, Fig. 29 in^35^). Figure 9 shows that, compared to both the above cases, the duck’s foot has a lower *C*_N,max_ but a relatively similar increase in d*C*_N_/d*α* up to *AoA* = 20°. While the delta wing and plate have a distinct stall at *AoA* =  ~ 33° the duck’s foot generates peak normal forces at ~ 55°. Some of these differences may be due to the higher Re number in the delta wing data and the asymmetric toes of the ducks. Another important difference is that of the leading-edge thickness. The vortex lift generated by delta wings depends not only on the swept leading edge but also on the sharpness of this leading edge^16^. In contrast, the duck’s foot has thicker digits at the leading edges and a thin webbed area. It can be seen that, unlike the delta wing and the thin plate, the duck’s foot has no distinct stall angle (Figs. 4, 9) making it functional at a wide range of *AoA*. Moreover, at *AoA* = 60° as the *C*_L_ starts to decrease the *C*_D_ approaches maximum (Fig. 4). As a result, the force normal to the foot (Fig. 9) and the resultant force (Figs. 3f, 4) remain high between 30° and 140°, making the foot a useful appendage for hydrodynamic propulsion regardless of the *AoA*. Both lift and drag are used for propulsion. The lift-to-drag ratio has a transition point at 60° and this transition is explained below using the flume measurements.

Despite its shape, the duck-foot model has distinct differences from the delta wing. First, we found no evidence of vortex generation on the suction side of the foot in the PIV data or in preliminary flow visualization attempts. Such a vortex, formed over the swept leading edge, is expected from delta wings^36,37^. Second, the flow pattern and observed foot kinematics (*AoA*) are very different from lift-dominated propulsion, and rather typical of a low aspect-ratio plate switching from lift production at low AoA to fully separated flow. The near-wake flow dynamics appears to change at an *AoA* higher than 60°. Above this angle, we observed variations in the mean and turbulent properties as well as in the flow patterns that developed. When applying POD to the velocity field, we would have expected to identify coherent structures^32^ at the intermediate modes (3:5) associated with turbulence, if such exist; these were only recognized for *AoA*s between 30° and 60°. Given the low aspect-ratio delta-like shape, coupled with relatively low Re number flow conditions, the flow evolved in the wake region exhibited a peculiar behavior in which the leading-edge region mostly dominated it. At this range of *AoA*, we obtained an organized symmetrical shedding aligned with high values of lift. This is in line with the Reynolds stress trends, where a sharp increase was measured above 60°. Above 60°, where the wake appears to be separated, the lift production decreases and drag peaks to maximum values (above 75°). At the higher modes (6:8) the energy content level is very low (< ~ 1% of the POD modes cumulative energy), rendering the information they hold to be of limited value.

Based on all the contained data, it seems that the foot of *A. nyroca* functions more as an oar than as a delta wing during vertical dives. A delta-shaped wing commonly augments lift through the formation of leading-edge vortices; formed at the sharp leading edges and causing flow reattachment to the wing after separation^38^. These vortices shed to the wake, generating a strong circulatory motion that enhances the lift over the wing. The fluid over the leading edge accelerates downward, resulting in an enhanced lift generation, termed a nonlinear or vortex lift^39^. This phenomenon has been observed also at Re numbers in the order of *O*(10^4^) at moderate *AoA* (i.e.: 20°)^40^. Here, we did not observe such an increase nor did we observe a strong circulatory motion shed to the wake, as determined from the POD analysis. Even when lift was produced at low *AoA* we did not observe the presence of leading-edge vortices, which is one of the defining features of a delta wing. We suggest that this is due to the thickness of the foot’s leading edge (as explained above) and the small dimensions of the foot. The length of the foot is relatively short compared with other cases in which a leading-edge vortex was identified over a delta wing. Due to the short chord and leading edge, the boundary layer over the foot is not developed and a leading-edge vortex cannot grow while moving along the short leading edge.

Over most of the flapping cycle the foot acts as an oar generating large normal forces that, according to the orientation of the webbed area (Figs. 1, 3), contribute to propelling the duck along its longitudinal axis and therefore downwards, against buoyancy and body drag. Our analysis focused on the steady hydrodynamic performance of the foot’s planform. The unsteady aspects of the paddling motion are beyond the scope of the present study. Acceleration-reaction and flow hysteresis are undoubtedly important and necessary for propulsion in the ducks. The reduced frequency of the vertically diving ducks (paddling frequency $\times$ the semi-chord/foot speed) was 0.18, indicating that unsteady effects cannot be ignored. However, such unsteady effects are hard to study in wind tunnels and flumes. Moreover, these unsteady effects are usually estimated and added to quasi-steady models that are based on steady flow data, as measured here. Figure 3 shows that during vertical diving the resultant force from the quasi-steady lift and drag is directed downwards throughout most of the power phase. Additional unsteady effects will be particularly important at the two transient stages of the power phase. At power phase initiation, acceleration reaction should augment downwards thrust as the feet accelerate upwards at large *AoA*. The opposite is expected at the end of the power phase, when the acceleration reaction from the decelerating feet should result in an upwards-directed force^27^. However, during the last 20% of the power phase the foot is moving medially relative to the body but also at low *AoA* relative to the water. Terminating a drag-based power stroke with medial motion is an efficient way to avoid the penalty of acceleration-reaction from the decelerating foot^41^. In addition, the medial movement is ideal for supplementing the forward propulsion force with lift, when the swimming speed is low. To achieve this, the ‘paddle’ should be able to generate lift despite its primary adaptation for drag production. Thus, the triangular webbed feet of the duck seem to offer a hydrodynamic compromise between a drag-producing oar and a lift-producing appendage.

## Methods

### Experiments with live ducks

**Figure 10:**
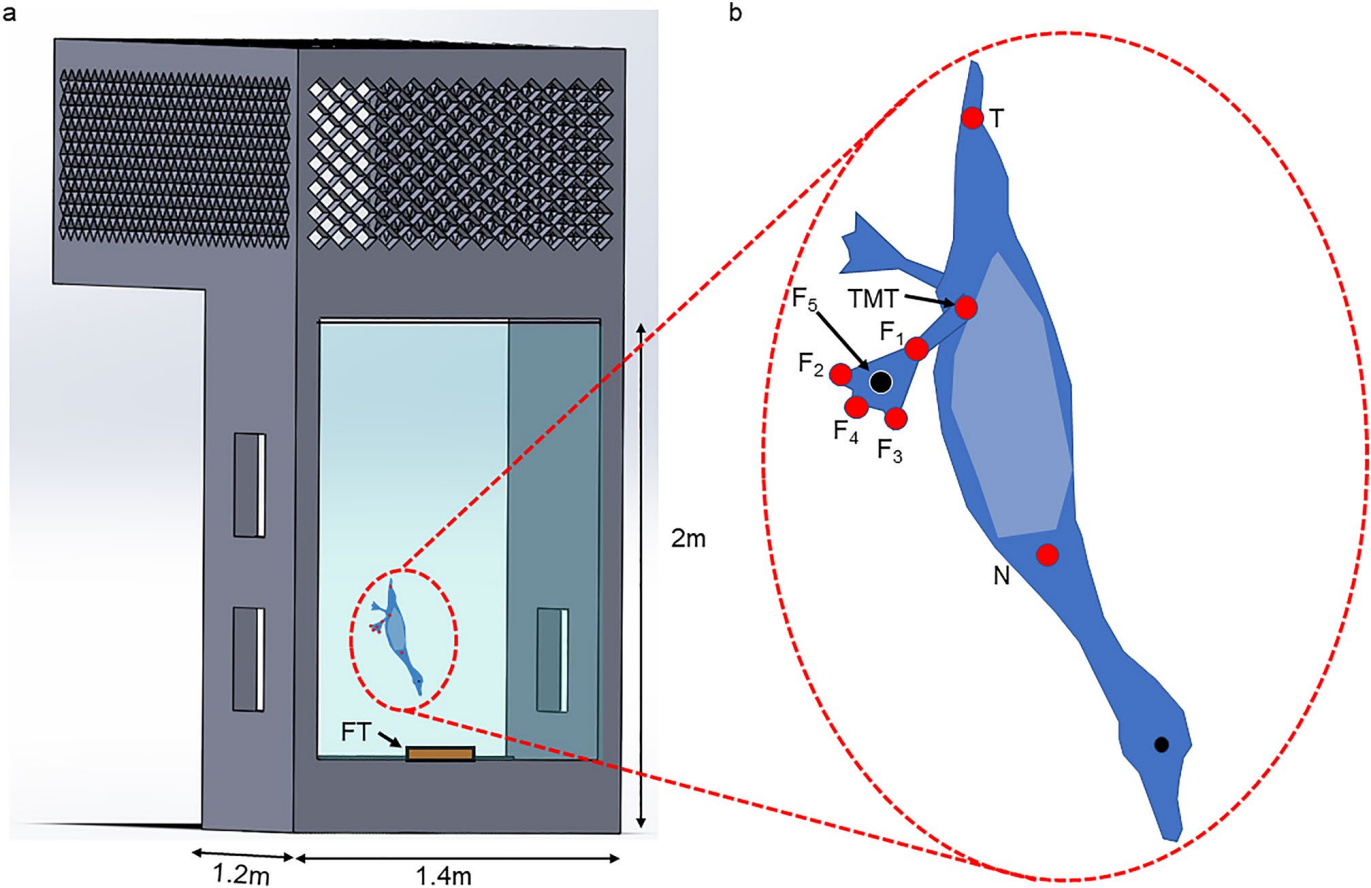
Extraction of paddling kinematics from live birds: (**a**) The birds were filmed descending the water column to a feeding tray (FT) at the bottom of a vertical diving tank. Three high-speed video cameras captured the paddling motion of the feet through the tank’s windows; (**b**) In the resulting movies we digitized landmarks on the duck’s body and foot to extract the foot’s kinematics, focusing on the angle-of-attack (*AoA*).

Live animal experiments were conducted under permits from the Tel Aviv University Ethics Committee for Experiment with Animals (permit: AU-LS-IL-220110402) and the Israel Nature and Parks Authority (permit: 2023/43,245). All methods were performed in accordance with the relevant guidelines and regulations and the study is reported in accordance with the ARRIVE guidelines. Three male and three female ferruginous pochards (*Aythya nyroca*) were trained to dive vertically for food in a large vertical diving tank (1.4m × 1.2m × 2m, Fig. 10a). We trained the birds by placing their food (lettuce and wheat grains) in a tray initially suspended just beneath the water surface and then lowered the tray gradually, forcing the birds to dive deeper each time to reach the food. Training lasted 2–3 days, after which the ducks were voluntarily diving from the surface to the feeding tray positioned on the bottom of the tank (2 m depth). The vertical swimming of the birds down to the tray was filmed through windows in the tank walls using three high-speed cameras (Fastcam SA3, Photron) filming at 250 frames per second. The cameras were synchronized by internal hardware and were spatially calibrated by moving a wand with two markers in the mutual field of view of all cameras^42^. The resulting movies displayed the vertical swimming of the birds as they reached 1.5 m depth.

### Extraction of paddling kinematics

Using the three camera views we digitized for each video frame the position of 7 markers on the body of the birds (Fig. 10b): the connection between the neck and body (‘N’), the base of the tail (‘T’), the tibia-metatarsus joints of the left and right legs (‘TMT_L_’ and ‘TMT_R_’, respectively), and three points on either the left or right foot: the inter-digit joint (‘F_1_’) and the tip of digits ii (‘F_2_’) and iv (‘F_3_’). Using the Matlab code DLTdv5^43^, we extracted the instantaneous 3D positions of these markers in each video frame and used the data to extract the foot trajectory and orientation during the power phase of the paddling cycle. From the data we calculated a fourth foot point (‘F_4_’) halfway between F_2_ and F_3_, and used it with F_1_ to define the foot’s central chord (bisector of the delta shaped planform). Using F_4_ and F_1_ we also calculated point ‘F_5_’ located at 2/3 of the central chord (Fig. 10b) representing the geometric center of delta wings^17^. The body points were used to determine the ducks’ swimming speed and orientation and to evaluate the paddling kinematics in the context of the body’s frame of reference^27^. The feet data were used to measure the instantaneous *AoA*s during the power phase of the paddling cycle. The foot velocity was found numerically^44^ from the time-varying position data of F_5_. The instantaneous *AoA* of the foot was calculated as the angle between the central chord and the foot velocity vector. To calculate this angle, we first shifted the origin of the coordinate system to point F_5_ and then rotated the foot data and velocity to align the foot’s central chord with the X axis. We then measured the angle between the rotated velocity vector and the central chord in the XZ plane. At the last 20% of the power phase the foot moves medially (see Results, Section “Paddling kinematics”), and for this duration the AoA is defined between the rotated velocity vector and the central chord in the XY plane.

### Foot model and scaling

**Figure 11:**
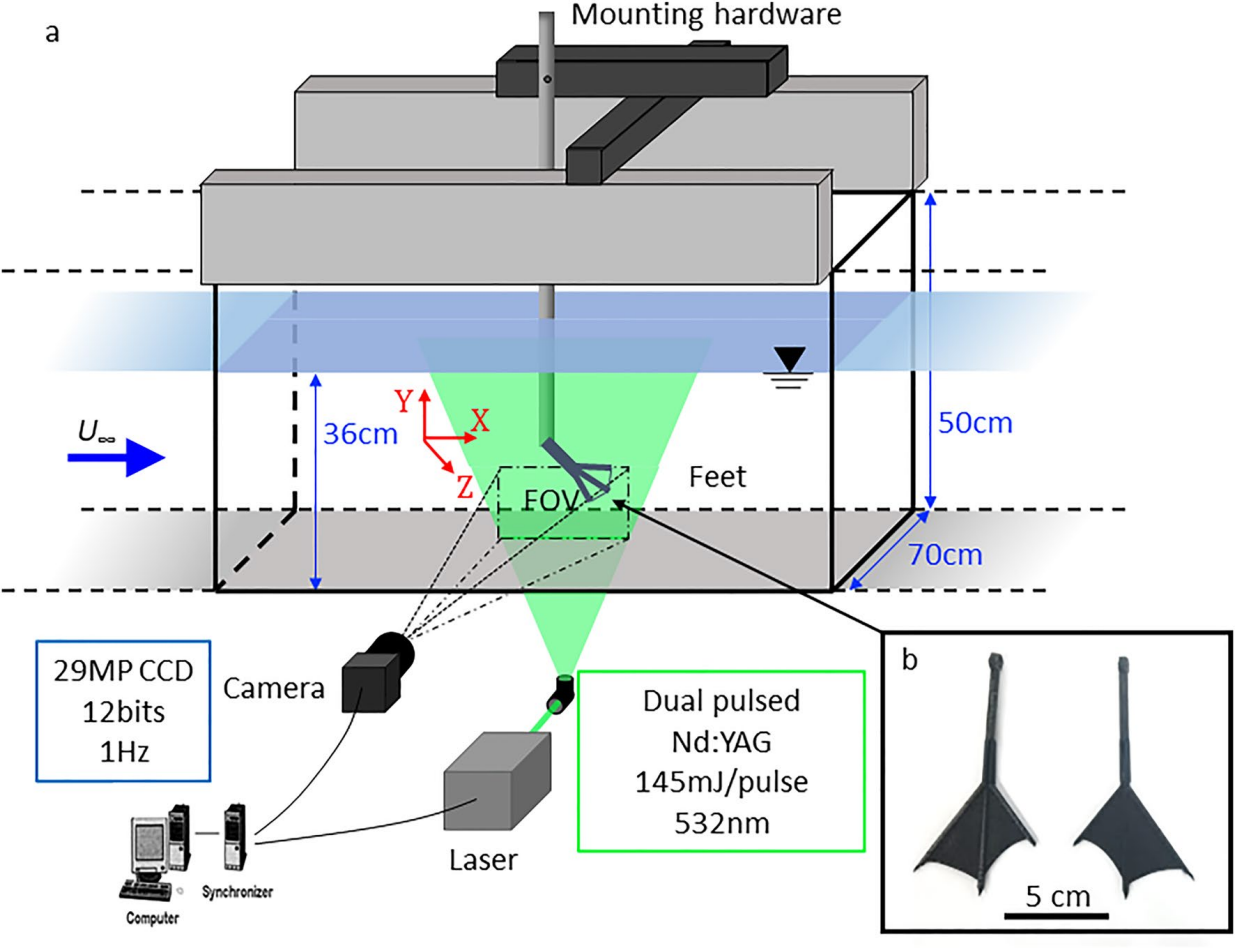
Set-up for flow analysis: (**a**) schematic illustration of the experimental set-up in the water flume and the PIV system (FOV—field of view); (**b**) 3D-printed *A. nyroca* feet models at 1:1 scale. Such models were used in the flume experiments, and a 2:1 model was used in the wind-tunnel experiments.

We used three *A. nyroca* mounted specimens obtained from the Steinhardt Museum of Natural History (Israel) to measure digit length, digit diameter, and inter-digit angles of the fully spread foot. We used the averaged morphometric data to construct 3D duck-foot models in Solidworks (Dassault Systems). A thin rod replaced the metatarsus and the computer 3D models were 3D printed (Onyx One, Markforged). We printed two physical foot models: one on a 1:1 scale (Fig. 11b) for the flume experiments; and the other one, magnified 2:1, for the wind tunnel study. All models were polished and smoothed on both sides to avoid rough surfaces.

### Wind tunnel experiments

The lift and drag coefficient of the duck-foot models as a function of their *AoA* were measured in a wind tunnel. The 3 m long tunnel had a 0.2 m × 0.21 m diamond-shaped cross-section. The models were tethered in the working section of the wind tunnel through their centroid and connected via a lever to a force measurement apparatus^45^ that measures forces in the vertical (hereafter ‘lift’) and horizontal (parallel to the free stream flow in the wind tunnel, hereafter ‘drag’) axes. The connection of the tether to the foot was through a rotating sleeve that allowed us to adjust the geometric *AoA* of the foot (-30°$\le$ *AoA* $\le$ 180° in steps of ~ 10°) without altering the position of the foot’s centroid in the wind tunnel. The geometric *AoA* in each step was set manually and verified to ± 1° by measuring the digital images taken by a horizontally levelled camera with its optical axis perpendicular to the tunnel. The weight of the foot model was subtracted from the vertical force (lift) measurement, and the drag of the tether without the foot was subtracted from the horizontal (drag) measurements. The wind speed was set to 5.2 m s^−1^. With the 2:1 scaling of the foot model the Re number during the experiments was 31,000 (using the central chord as the characteristic length). Thus, the experiments simulated the flow regime of a real-size duck foot moving through water at 0.7 m s^−1^. This Re number was considered typical of foot propulsion during vertical descent, since the measured mean (± SD) speed of the foot during the power phase was 0.73 ± 0.18 m s^−1^ in our live duck experiments (see Section “Results”).

### Force estimation from movies

The direction of the quasi-steady resultant hydrodynamic force (lift + drag) generated by the foot during the power phase (Fig. 3f) was estimated using the webbed foot area (*A* = 19 × 10^–3^ m^2^) measured from a photograph. Using the foot’s AoA and Fig. 4 we found the lift and drag coefficients (*C*_*L*_ and *C*_*D*_, respectively) for each movie frame of the power phase. The instantaneous speed of the foot relative to water (*U*) was measured at F_5_ (from Section “Extraction of paddling kinematics”). The magnitude of the instantaneous resultant force (*F*) is:

$$F= \frac{1}{2}\rho A{U}^{2}\sqrt{{C}_{L}^{2}+{C}_{D}^{2}}$$

where $\rho$ is water density (1,000 kgm^-3^). The direction of this force in 3D space is found after rotating the foot data and velocity so that the central chord is aligned with the X axis (Section “Extraction of paddling kinematics”). Lift is perpendicular to the velocity vector (according to the AoA) and drag is in the opposite direction of velocity. The resultant force is rotated relative to the lift by $\alpha ={\mathrm{tan}}^{-1}\frac{{C}_{D}}{{C}_{L}}$. Once the direction of the instantaneous resultant force is found, it is simply rotated with the foot data back to the original foot orientation. The magnitude and direction of the instantaneous resultant force can then be plotted in in the cameras’ frame of reference as quiver plots (Fig. 3f).

### Water flume set-up and PIV experiments

The interaction between the duck’s foot and the surrounding fluid as a function of *AoA* was studied from measurements of the flow field developed at the near-wake of the foot. The experiments were performed in an open channel flow (flume) using 2D-PIV. The Re number, based on the mean current speed in the flume and the chord of the duck’s foot, was 10,000. The flume cross-sectional dimensions were 0.7 m × 0.5 m, with a trough length of 15 m, where middle 5 m comprised transparent glass. A centrifugal pump recirculated the water through two large reservoirs located at the entrance and exit of the flume. The water height was 0.36 m in the flume to ensure that the submerged duck-foot model was away from both the surface and bottom boundary layer flow effects. The uniform flow velocity within the channel was calculated as *U*_0_ = *Q*/*A* = 0.19 m s^−1^, where *Q* is the flow rate measured by a rotary flow meter and *A* is the wetted cross-sectional area of the water in the flume. The flow field measurements were performed using particle image velocimetry (PIV)^46^ in the streamwise-normal plane. The experimental set-up is depicted in Fig. 11a, where the duck-foot model was placed in the flume and the flow over the foot was imaged by illuminating a plane of light from beneath the channel. The measurement location was set at 0.25 the span and 0.5C downstream of the foot model (C = foot’s central chord length, 0.05 m) encompassing an area of ~ 7C horizontally and ~ 5C vertically. The PIV system comprised a dual pulsed Nd:YAG laser (Quantel Inc. EverGreen145) emitting a 145 mJ/pulse at a wavelength of 532 nm. The light sheet was approximately 1 mm thick and the particles constituted fused borosilicate glass microspheres, 11 µm average diameter (Potters Industries). A 200 mm camera lens with a 20 mm extension tube was mounted on a 29 MP double exposure CCD camera (TSI: PowerView™) with a dynamic range of 12-bits operating at 1 Hz. The camera yielded a field of view of 38 cm × 28 cm. Each experiment comprised 400 image pairs in order to allow for statistical convergence. The time delay between each two laser pulses was set to 2 ms. Each image pair was analyzed using cross-correlation (Insight 4G™, TSI Inc.) to estimate the two velocity components in the streamwise (*u*) and wall-normal (*v*) directions over the two-dimensional plane (*x* and *y*). Each image pair was divided into interrogation regions of 64 × 64 pixel^2^, with 50% overlap for the cross-correlation, and vector maps were subsequently filtered for outliers using both local and global filters. Uncertainty for the instantaneous velocities was estimated as 2–4% of the measured value, and an uncertainty of 8–10% was estimated for the computed instantaneous velocity gradients.

### Supplementary Information

Supplementary Figure 1.

## Data Availability

Paddling kinematics, force measurements and flow maps are available on CCU cluster domain https://ci.coastal.edu.

## List of symbols

$\rho$: Fluid density [kg m^−^^3^]
${\Omega }_{2}$: Mean spanwise vorticity [rad/s^−^^1^]
*AoA,*$\alpha$: Geometric angle-of-attack [°]
$A$: Webbed area of the foot [m^2^]
${A}_{flume}$: Wetted cross-sectional area of the flume [m^2^]
$b$: Paddling amplitude in the XY plane [m]
$C$: The chord of the foot [m]
*C*_*L*_: Lift coefficient
*C*_*D*_: Drag coefficient
*C*_*F*_: Aerodynamic force coefficient
*C*_*N*_: Normal force coefficient
$f$: Paddling frequency [Hz]
$$F$$: Hydrodynamic force [N]
*Q*: Flow rate in the flume [m^3^ s^−^^1^]
$T$: Power phase duration [s]
$\widehat{t}$: Non-dimensional time within a single power phase (0 $\le \widehat{t}\le$ 1.0)
$$U$$: Flow speed [m s^−^^1^]
${U}_{\infty }$: Free-stream flow speed [m s^−^^1^]
$${U}_{swim}$$: Swimming speed [m s^−^^1^]
$$St$$: Strouhal number
Re: Reynolds number
(X,Y,Z): Inertial cartesian coordinate system where Z is the vertical (positive end pointing up) [m]
(Xb, Yb, Zb): Cartesian coordinate system fixed on the duck’s body (see Fig. 1d) [m]
($$x,y,z)$$: Cartesian system of the flume. $$x$$ is in the streamwise direction (see Fig. 11) [m]
$(u,v,w)$: Instantaneous velocity components along the flume’s $x,y,z$ axes. $u$ is the streamwise velocity [ms^-1^]
$(u{\prime},v{\prime},w{\prime})$: Velocity fluctuation components [ms^-1^]
$(\overline{u },\overline{v },\overline{w })$: Mean velocity components along the flume’s $x,y,z$ axes [ms^-1^]
${A}_{i},{b}_{i}, {\varphi }_{i}$: Curve fitting parameters for the sum of sine function ${\sum }_{i=1}^{n}{A}_{i}\mathrm{sin}\left({b}_{i}\alpha +{\varphi }_{i}\right)$ relating the aerodynamic force coefficients with the angle of attack $\left(\alpha \right)$ of the foot
$$\overline{{u }{\prime}{v}{\prime}}$$: Reynolds stress [m^2^s^-2^]
TKE,$k$: 2D turbulent kinetic energy, TKE= $0.5\left(\overline{{u }^{{\mathrm{^{\prime}}}^{2}}}+\overline{{v }^{{\mathrm{^{\prime}}}^{2}}}\right)$ [m^2^s^-2^]
POD: Proper orthogonal decomposition
PIV: Particle image velocimetry
