# Supplementary material for: The hydrodynamic performance of duck feet for submerged swimming resembles oars rather than delta-wings
Source: Sci Rep. 2023 Sep 27;13:16217. doi: 10.1038/s41598-023-42784-w (PMC10533810; doi:10.1038/s41598-023-42784-w)
Supplement: Supplementary file 1 — Supplementary Figure 1. [file 41598_2023_42784_MOESM1_ESM.docx]

**Supporting Information**

The energy accumulation of the decomposed POD modes per flume (*AoA*) experiment on a semi-log figure, where the assumption is that, for each experiment, there is 100% energy that is distributed over the modes. The total energy contained by the modes is the highest at 15^o^ and then decreases up to 90^o^ where the trends flip when the angles become greater than 90^o^


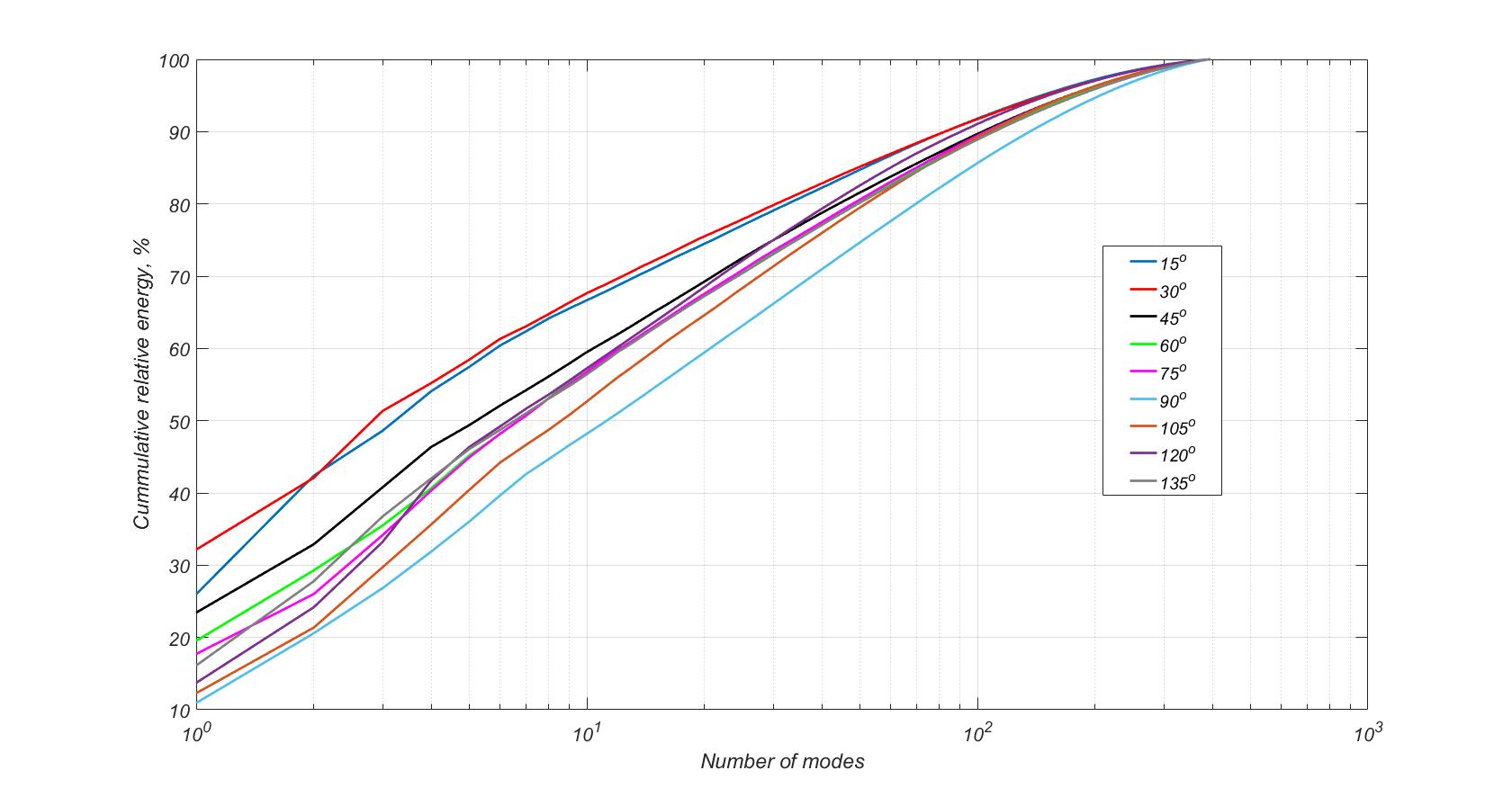


**Figure SI-1**: Cumulative kinetic energy (% out of 100) of the decomposed modes as a function of *AoA*. A semi-log is introduced to show the differences in the energy levels.
